# Supplementary material for: Characterising Eastern Grey Kangaroos (Macropus giganteus) as Hosts of Coxiella burnetii
Source: Microorganisms. 2024 Jul 19;12(7):1477. doi: 10.3390/microorganisms12071477 (PMC11279116; doi:10.3390/microorganisms12071477)
Supplement: Supplementary file 1 [file microorganisms-12-01477-s001.zip › Figure S1.pdf]

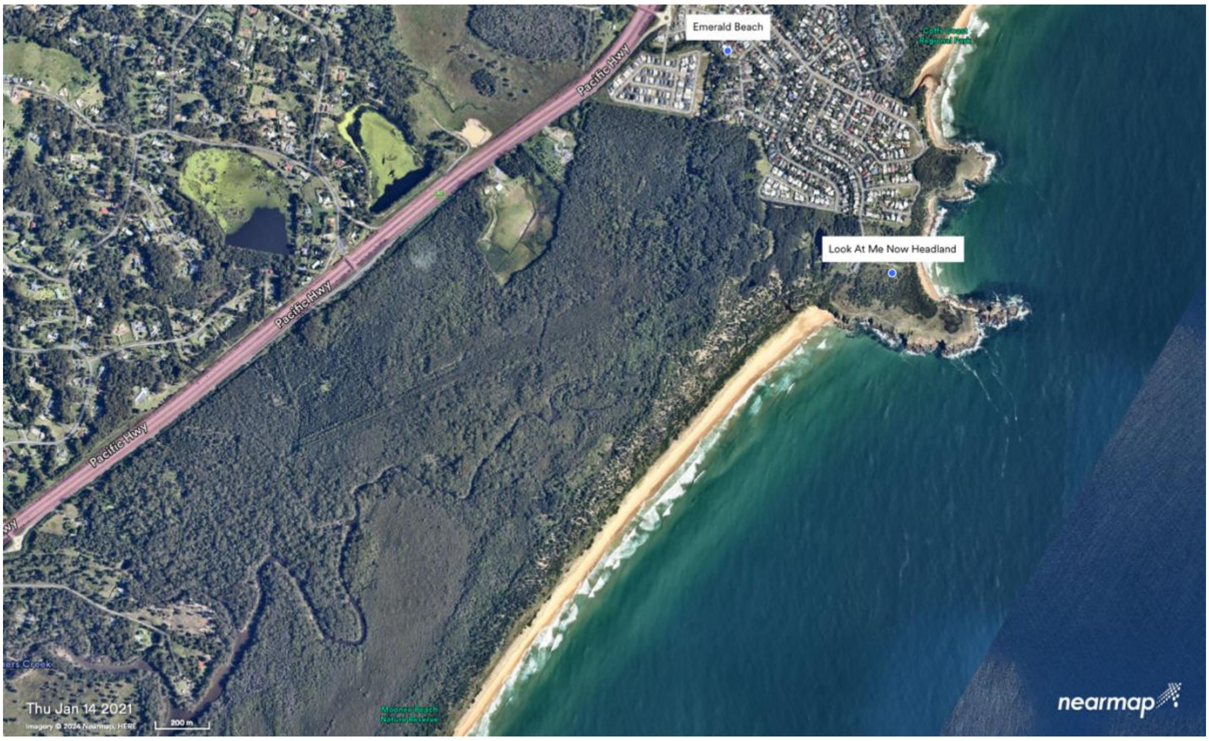

Figure S1: Map of Look At Me Now Headland and the surrounding area. The headland and study site (denoted) is bordered by coastline to the east, Emerald Beach township to the north, the Pacific Highway to the west and Moonee Beach Nature Reserve to the south. Map obtained from Nearmap Pty Ltd, Australia.
