# Supplementary material for: Characterising Eastern Grey Kangaroos (Macropus giganteus) as Hosts of Coxiella burnetii
Source: Microorganisms. 2024 Jul 19;12(7):1477. doi: 10.3390/microorganisms12071477 (PMC11279116; doi:10.3390/microorganisms12071477)
Supplement: Supplementary file 1 [file microorganisms-12-01477-s001.zip › Table S1.pdf]

Table S1: Primers and probes used in real-time PCR reactions in this study.

Gene	Primer/ probe	Sequence (5′ – 3′)	Ampli-con size (bp)	Final conc. (nM)	Ref.
IS*1111*	IS1111-F	AAAACGGATAAAAAGAGTCTGTGGTT	70	300	[31]
	IS1111-R	CCACACAAGCGCGATTCAT		300	
	IS1111-P	Quasar670[a]-AAAGCACTCATTGAGCGCCGCG-BHQ2[b]		150	
com1	com1-F	AAAACCTCCGCGTTGTCTTCA	76	400	[32]
	com1-R	GCTAATGATACTTTGGCAGCGTATTG		400	
	com1-P	FAM[c]-AGAACTGCCCATTTTTGGCGGCCA-BHQ1[d]		200	
htpAB	htpAB-F	GTGGCTTCGCGTACATCAGA	114	400	[33]
	htpAB-R	CATGGGGTTCATTCCAGCA		400	
	htpAB-P	FAM[c] -AGCCAGTACGGTCGCTGTTGTGGT -BHQ1[d]		200	
lin02483	lipHQ-F	AACCGGGCCGCTTATGA	62	50	[34]
	lipHQ-R	CGAACGCAATTGGTCACG		50	
	lipHQ-P	HEX-TTCGAATTGCTAGCGGCACACCAGT -BHQ1[d]		100	

[a] Quasar 670 carboxylic acid
[b] Black Hole Quencher-2
[c] 6-Carboxyfluorescein
[d] Black Hole Quencher-1
