# Supplementary material for: Characterising Eastern Grey Kangaroos (Macropus giganteus) as Hosts of Coxiella burnetii
Source: Microorganisms. 2024 Jul 19;12(7):1477. doi: 10.3390/microorganisms12071477 (PMC11279116; doi:10.3390/microorganisms12071477)
Supplement: Supplementary file 1 [file microorganisms-12-01477-s001.zip › Table S2.pdf]

Table S2: Signalment and summary of pathological, serological and molecular results for necropsied eastern grey kangaroos sampled at Look At Me Now Headland, NSW in 2021.

ID	Signalment	Histopathology findings	Serology titre Phase I	Phase II	PCR +ve tissues
309	Subadult female	Mild to moderate gastritis and enteritis; splenic granulocytosis; reactive lymph nodes*; mild biliary hyperplasia; mild alveolar histiocytosis.	1:4096	1:512	Kidney, gastro-intestinal tract, mediastinal lymph node, faeces
339	Adult female	Mild cardiac and pulmonary perivascular oedema; serous atrophy of peri-pancreatic fat; pancreatic zymogen granule depletion; splenic and lymph node* reactive lymphoid hyperplasia; mild lymphocytic hepatitis with mild hepatocellular necrosis; moderate chronic lymphoplasmacytic and eosinophilic gastritis, and enterocolitis with occasional degenerate parasites (presumed *Eimeria* spp.).	1:4096	1:1024	Bladder
386	Subadult male	Mild alveolar histiocytosis and pulmonary oedema; splenic and lymph node* reactive lymphoid hyperplasia; mild to moderate, chronic lymphocytic and granulocytic enterocolitis.	1:4096	1:512	Bladder, skeletal muscle
1121-01	Adult female	Locally extensive, acute granulocytic peritonitis and ureteritis; pulmonary oedema; splenic lymphoid hyperplasia, serous atrophy of fat; focal, chronic renal infarct with mild capsulitis and capsular fibrosis, interstitial nephritis, moderate cortical tubular ectasia and proteinosis; focal tracheal cartilage disruption with local mild chronic tracheitis and fibrosis; moderate eosinophilic and lymphoplasmacytic gastritis and enterocolitis; reactive, draining lymph nodes*.	1:8129	1:1024	None
1121-02	Subadult male	Splenic and lymph node lymphoid hyperplasia; multifocal, moderate chronic splenic granulomas with associated	1:128	< 1:32	Epididymis, gastrointestinal tract,

		vasculitis and microfilaria; pulmonary oedema and haemorrhage; focal, mild perivascular histiocytic and granulocytic interstitial pneumonia; multifocal, mild hepatitis with mild periportal fibrosis; multifocal, mild, chronic interstitial nephritis; mild cardiac perivascular oedema; mild to moderate gastritis and enterocolitis with intraluminal nematode larvae.			mesenteric lymph node, faeces, nasal swab and urogenital tract swab
1121-03	Subadult male	Chronic, mild, multifocal lymphoplasmacytic and histiocytic interstitial and tubular nephritis with occasional intratubular protozoa (*Klossiella* spp. presumptive); multifocal, mild membranous glomerulopathy; focal, mild, chronic pyogranulomatous hepatitis; multifocal, mild, chronic portal hepatitis with sinusoidal neutrophilia perivascular cardiac oedema; mild alveolar histiocytosis with pulmonary oedema and emphysema; reactive draining lymph nodes*; moderate, chronic granulocytic enterocolitis and with intralesional nematode parasites.<br><br>IHC negative on all tissues.	1:4096	1:512	Bladder, gastro-intestinal tract, heart, kidney, spleen, liver, skeletal muscle, mediastinal lymph node, nasal swab and urogenital tract swab
1121-04	Subadult male	Multifocal, mild chronic membranous glomerulopathy with tubular dilation and attenuation and lymphoplasmacytic cortical Interstitial nephritis; pulmonary oedema and mild alveolar histiocytosis focal pulmonary vascular thrombosis with perivascular inflammation; splenic lymphoid hyperplasia; serous atrophy of fat; moderate cortical lymphoid depletion of lymph nodes*; moderate, chronic gastritis and enterocolitis with intraproprial protozoa (*Eimeria* spp., presumptive).	< 1:32	< 1:32	Mediastinal lymph node, whole blood, nasal swab, faeces
1121-05	Adult female	Multifocal, mild interstitial histiocytic and granulocytic tubular nephritis; focal, mild, acute necrosuppurative	1:4096	1:512	Lung, skeletal muscle, gastro-intestinal tract,

		hepatitis; pulmonary oedema, emphysema and haemorrhage with mild alveolar histiocytosis; splenic and lymph node* reactive lymphoid hyperplasia; splenic stromal spindle cell proliferation; serous atrophy of fat; mild to moderate lymphoplasmacytic and granulocytic gastritis and enterocolitis, with intramucosal larval nematodes; perivascular cardiac oedema.			ovary, urogenital tract swab
1121-06	Adult female	Mild granulocytic portal hepatitis with mild bile duct hyperplasia; multifocal, mild to moderate, chronic lymphoplasmacytic interstitial and tubular nephritis; splenic and lymph node* reactive lymphoid hyperplasia; moderate eosinophilic and lymphoplasmacytic gastritis and enterocolitis; serous atrophy of fat; localised inflammation of connective tissue associated with skeletal muscle.	1:2048	1:512	None
1121-07	Subadult female	Splenic lymphoid hyperplasia; mild interstitial and tubular nephritis with mild perivascular oedema; mild to moderate, chronic lymphoplasmacytic and granulocytic enterocolitis; reactive and oedematous lymph nodes*; serous atrophy of fat.	1:16384	1:2048	Liver
1221-01	Subadult male	Multifocal, mild, chronic granulomatous hepatitis with portal fibrosis and intralesional degenerate trematode eggs (*Fasciola* spp., presumptive); focal, mild, chronic cholangiohepatitis; pulmonary oedema and mild alveolar histiocytosis; pericardial oedema and mild inflammation; perivascular oedema; multifocal, mild, chronic interstitial nephritis; mild membranous glomerulopathy; reactive lymph nodes*; moderate chronic granulocytic and lymphoplasmacytic gastritis and enterocolitis with intramucosal larval nematodes and protozoa; focal, mild, chronic gastric ulcer; multifocal, moderate, chronic lymphoplasmacytic epididymal	1:128	< 1:32	Epididymis

		perivasculitis and vasculitis with medial arteriosclerosis. IHC negative.			
1221-02	Adult male	Multifocal, mild, chronic lymphoplasmacytic portal hepatitis with fibrosis; bile duct hyperplasia and capsular fibrosis; perivascular pulmonary oedema; multifocal, mild, chronic interstitial and tubular nephritis with intratubular protozoa (*Klosiella* spp. presumptive); moderate chronic lymphoplasmacytic and granulocytic enterocolitis with occasional intramucosal larval nematodes; reactive lymph nodes*; segmental, mild, chronic subendocardial fibrosis; locally extensive, mild, perinodal and peripancreatic peritonitis.	1:4096	1:256	Liver

* Sampled lymph nodes included mediastinal, mesenteric, axillary and inguinal but they were not differentiated during processing.
